# Using Social Media in Health Care Research Should Proceed With Caution. Comment on “The Use of Social Media for Health Research Purposes: Scoping Review”

**DOI:** 10.2196/35286

**Published:** 2022-01-28

**Authors:** Abdias Girardi, Nikhi Paul Singh, Carter Joseph Boyd

**Affiliations:** 1 Heersink School of Medicine University of Alabama at Birmingham Birmingham, AL United States; 2 Hansjörg Wyss Department of Plastic Surgery New York University Langone Health New York, NY United States

**Keywords:** public health, epidemiology, research, health, medical, social networking, infodemiology, eHealth, text mining, medical education, social media, information technology, health care, HIPAA, education

We thank Bour and colleagues for their recent publication regarding the use of social media for health research purposes [1]. Many people use social media to gain knowledge about medicine, and as such, it is important to characterize the reliability of research health topics on these platforms.

A major advantage of social media is its use as an effective vehicle for large-scale dissemination of information, as seen throughout the COVID-19 pandemic. In addition to facilitating health surveillance and disseminating public health information, social media has been helpful in providing social support and behavioral counseling for people in need of such services [2]. Lack of transportation is often a barrier to health care access, and although not every medical condition can be addressed online, access to social media may assist in health education.

Social media has limitations though. Ethical issues concerning consent, privacy, and confidentiality of users are commonly encountered. Social media is considered to be public, and user consent is not provided while collecting social media data. Health care professionals worry about breaching patient confidentiality and facing consequences under the federal Health Insurance Portability and Accountability Act (HIPAA) and state privacy laws. Although it is acceptable to share deidentified patient information, a study of medical blogs proved that keeping patient information deidentified might not be as easy as it seems—individual patients were described in 42% of the 271 samples studied [3]. In this cohort, 17% of cases were found to include enough information for patients to identify themselves or their providers [3]. Moreover, social media users have a skewed distribution toward adolescents and young adults. Thus, the representativeness of the sample may be misleading, resulting in biased findings and preventing generalization to the entire population [1].

While social media has the potential to be a useful tool in health research, both factual and false information can be posted online. Currently, no verification system exists for health information on these platforms. Users can be exposed to dangerous and fake content posted by detractors and chatbots on social media. A recent study from the Massachusetts Institute of Technology demonstrated that false claims are 70% more likely to be retweeted on Twitter than the truth [4]. This represents a serious threat to public health, as misinformation can be readily spread.

Lack of regulation in social media poses a potential risk to patient safety and public well-being. The American Medical Association (AMA) *Journal of Ethics* provides guidance for physicians navigating these platforms and emphasizes the convection of truthful information and confrontation with misleading or false information [5]. The use of a systematic approach and adherence to the AMA’s guidelines may prove useful, preventing physicians from accidentally sharing patient information or misinformation. Social media holds great promise in medicine, but caution should be taken.

## Abbreviations

AMA: American Medical Association
HIPAA: Health Insurance Portability and Accountability Act
